# Left atrial functional assessment and mortality in patients with severe aortic stenosis with sinus rhythm

**DOI:** 10.1186/s12947-020-00231-0

**Published:** 2021-01-02

**Authors:** João Ferreira, Valdirene Gonçalves, Patrícia Marques-Alves, Rui Martins, Sílvia Monteiro, Rogério Teixeira, Lino Gonçalves

**Affiliations:** 1 Serviço de Cardiologia, Centro Hospitalar e Universitário de Coimbra, Praceta, R. Prof. Mota Pinto, 3004-561 Coimbra, Portugal; 2 Departamento Cardio-torácico, Clínica Girassol, Luanda, Angola; 3 Faculdade de Medicina, Universidade de Coimbra, Coimbra, Portugal; 4 iCBR, Coimbra Institute for Clinical and Biomedical Research, Universidade de Coimbra, Coimbra, Portugal

**Keywords:** Aortic stenosis, Atrial mechanics, Echocardiography, Biomarker, Prognosis

## Abstract

**Background:**

Aortic valve stenosis (AS) is the most common primary valvular heart disease leading to surgical or percutaneous aortic valve replacement (AVR) in Europe and its prevalence keeps growing. While other risk factors in severe AS are well documented, little is known about the prognostic value of left atrial (LA) function in AS. Our aim is to clarify the relationship between LA function measured at severe AS diagnosis (evaluated by means of volumetric assessment) and all-cause mortality during follow-up.

**Methods:**

We retrospectively evaluated patients diagnosed with severe AS for the first time at our echocardiography laboratory. We evaluated LA reservoir, conduit and pump function by measuring LA volumes at different timings of cardiac cycle. Treatment strategy was decided according to heart team consensus and patient decision. We divided patients into groups according to terciles of LA reservoir, conduit and pump function. Primary outcome was defined by the occurrence of all-cause mortality during follow-up.

**Results:**

A total of 408 patients were included in the analysis, with a median follow-up time of 45 months (interquartile range 54 months). 57.9% of patients underwent AVR and 44.9% of patients registered the primary outcome during follow-up. Left atrial emptying fraction (LAEF) was the best LA functional parameter and the best overall parameter in discriminating primary outcome (AUC 0.845, 95%CI 0.81–0.88, *P* < 0.001). After adjustment for clinical, demographic and echocardiographic variables, cumulative survival of patients with LAEF < 37% and LAEF 37 to 53% relative to patients with LAEF ≥54% remained significantly lower (HR 13.91, 95%CI 6.20–31.19, *P* < 0.001 and HR 3.40, 95%CI 1.57–7.37, *P* = 0.002, respectively). After adjustment for AVR, excess risk of LAEF < 37% and LAEF 37 to 53% relative to LAEF ≥54% remained significant (HR 11.71, 95%CI 5.20–26.40, *P* < 0.001 and HR 3.59, 95%CI 1.65–7.78, *P* = 0.001, respectively).

**Conclusions:**

In patients with a first diagnosis of severe AS, LA function, evaluated by means of volumetric assessment, is an independent predictor of all-cause mortality and a more potent predictor of death compared to classical severity parameters. These data can be useful to identify high-risk patients who might benefit of AVR.

**Supplementary Information:**

The online version contains supplementary material available at 10.1186/s12947-020-00231-0.

## Introduction

Aortic valve stenosis (AS) is the most common primary valvular heart disease leading to surgical or percutaneous valve replacement in Europe and its prevalence keeps growing due to the generalized aging of population [1]. According to current recommendations, aortic valve replacement (AVR) is indicated in the presence of symptoms and/or left ventricular systolic dysfunction [left ventricular ejection fraction (LVEF) < 50%] [2]. However, both symptoms and systolic dysfunction can appear late in the course of the disease, being often synonym of irreversible damage to the myocardium when found [3]. On the other hand, structural changes can occur before symptom onset and are associated with worse prognosis, even after AVR [4, 5]. With the increasingly widespread access to both surgical or transcatheter AVR, there is a necessity to find other sensitive markers present at an earlier stage of the disease.

In patients with AS, there is a background of chronically increased left ventricular afterload which is associated with structural and functional changes in the left atrium (LA). As such, LA enlargement is a common finding in these patients [6] and correlates with increased left ventricular filling pressures [7]. Also, the ongoing pressure overload leads to disturbance in the three functional phases of LA (reservoir, conduit and contractile phase [8]), particularly in the contractile phase. Reservoir and conduit phase impairment also seems to occur but may be a late finding, more commonly associated with pulmonary hypertension [9].

LA functional assessment has been performed by means of volumetric method in a number of diseases, such as dilated cardiomyopathy [10], atrial fibrillation (AF) [11, 12], ventricular arrhythmias [13] and heart failure (HF) [14]. While much is known about LA structural damage as a predictor of mortality in different diseases such as dilated cardiomyopathy [15], myocardial infarction [16], mitral regurgitation [17] and more recently in AS [18], there is limited information regarding LA function as a predictor of prognosis in patients with AS.

Although there is recent data regarding the impact of LA mechanics, evaluated by speckle tracking echocardiography, in the outcome of patients with AS [19–21], the lack of standardisation between vendors and lack of validation for its use in thin-walled chambers should be taken into account when using this technique. Three-dimensional echocardiography has also been proved to be more strictly correlated to LA dimensions measured by cardiac magnetic resonance [22]. However, it is not available in every echocardiography laboratory and still has important issues with temporal resolution. Therefore, two-dimensional LA volumetric assessment remains a simple, reproducible and the routinely used method to measure LA function.

Thus, our primary aim is to assess the LA function, evaluated by means of volumetric assessment, in patients with severe AS diagnosis, and to study its potential impact on all-cause mortality during follow-up.

## Methods

### Study participants

We retrospectively evaluated 667 patients diagnosed with severe AS [V_max_ ≥ 4 m/s, mean pressure gradient (MPG) ≥ 40 mmHg, or aortic valve area (AVA) ≤ 1.0 cm^2^] for the first time at our echocardiography laboratory, between December 2010 and February 2020. The following patients were excluded: (1) those with prosthetic valves, previous cardiac surgery, congenital heart disease, supravalvular or subvalvular AS or dynamic left ventricular outflow tract (LVOT) obstruction; (2) those with mitral stenosis (defined as functional mitral valve area ≤ 2.5 cm^2^; (3) those with AF at index echocardiogram or with previous history of AF; (4) those with poor acoustic window with suboptimal imaging of LA and (5) those with severe concomitant valvular lesions. Clinical, analytical and demographic baseline characteristics, including presence of coronary artery disease (CAD), cardiovascular risk factors, functional status, normal cognition and other comorbidities were collected from the hospital medical records.

The study was approved by the institutional scientific and bioethical committees and was performed in accordance with the Declaration of Helsinki.

### Echocardiographic measurements

All patients underwent comprehensive 2-dimensional and Doppler echocardiographic evaluation. Exams were conducted using a General Electric Vivid S6 or Vivid 7 Ultrasound system and 3Sc-RS tissue harmonics transducer operating with a frequency of 1.2/3.4 MHz. Obtained images were then exported to a computer database and processed with EchoPAC® Clinical Workstation Software version 113 (General Electric, Healthcare, Chicago, Illinois). AVA was calculated using the continuity equation [23], and indexed AVA (AVAi) was obtained after indexing AVA to body surface area (BSA). LVOT diameter was measured in parasternal long-axis view in accordance with recent recommendations [24]. LVOT velocity-time integral (VTI) was measured in apical 5-chamber view. V_max_ and MPG were obtained using continuous-wave Doppler in the view in which cursor alignment with flow was the most appropriate, recording the highest value of V_max_ and aortic VTI. All pressure gradients were calculated using simplified Bernoulli equation. Very severe AS was defined when MPG was ≥60 mmHg or V_max_ ≥ 5 m/s. LVEF was measured with the Simpson biplane method. Wall thickness was measured at end-diastole in parasternal long-axis view. Left ventricular hypertrophy (LVH) was defined when left ventricular mass was > 115 g/m^2^ for men and > 95 g/m^2^ for women, measured by 2D-directed M-mode. LV diastolic function was assessed by measurement of mitral E velocity, mitral A velocity, mitral E/A ratio, mitral “L” velocity, pulsed-wave tissue Doppler-derived mitral annular early diastolic velocity measured at septum (septal e’), mitral E/e’ ratio, indexed LA maximum volume and tricuspid regurgitation velocity, in accordance to American Society of Echocardiography guidelines [25]. Valvular regurgitation severity was assessed following recommendations from European Society of Cardiology [26]. For each measurement, at least three cardiac cycles were averaged.

### Left atrial volumetric assessment

The 3 LA functional phases can be evaluated non-invasively with echocardiography. For that effect, LA volumes were measured using the biplane method of disks (modified Simpson) in apical 2- and 4-chamber views, at different phases during the cardiac cycle, with the method described by O’Connor et al [8]. We measured the following LA volumes: (1) maximal LA volume (Vol_max_), during ventricular end-systole right before mitral valve opening; (2) minimum LA volume (Vol_min_), during ventricular end-diastole right after mitral valve closure and (3) Vol_preA_, just before the start of the “P” wave on electrocardiogram. We excluded left atrial appendage and pulmonary vein orifices from measurement. LA reservoir function was evaluated by measuring LA total emptying volume (Vol_max_ - Vol_min_) and LA emptying fraction (LAEF) [(LA total emptying volume/Vol_max_) × 100]. LA conduit function was evaluated by measuring LA passive emptying volume (Vol_max_ - Vol_preA_) and LA passive emptying fraction (LAPEF) [(LA passive emptying volume/Vol_max_) × 100]. LA pump function was evaluated by measuring LA active emptying volume (Vol_preA_ - Vol_min_) and LA active emptying fraction (LAAEF) [(LA active emptying volume/Vol_preA_) × 100]. All volumes were then indexed for BSA.

### Treatment decision and follow-up

Treatment strategy (conservative, percutaneous, surgical or conservative followed by percutaneous or surgical) was decided according to heart teams’ consensus and patient decision. The majority of study patients were diagnosed and followed-up in outpatient setting at our center. Other patients were diagnosed with AS after hospital admission, and then followed up in outpatient setting. A minority of patients were diagnosed with AS after hospital admission and underwent surgery during the same hospitalization. Information on follow-up was obtained by assessing patient registries at our institution. The end-point was overall survival after diagnosis, with conservative, percutaneous or surgical treatment. During follow-up patients were regularly observed by their assigned cardiologist. Decisions regarding treatment were made in heart team consensus, always with the approval of patient’s cardiologist and according to patient’s will.

### Statistical analysis

Study population was divided into groups according to terciles of LA functional assessment (LAEF, LAPEF and LAAEF). Normality of continuous variables was evaluated by histogram observation and Kolmogorov-Smirnov test. Continuous variables are presented as mean ± standard deviation (SD) or median with interquartile range (IQR), as adequate, and categorical variables as frequencies or percentages. Group comparison was made with Student *t*, Wilcoxon rank-sum, unidirectional ANOVA or Kruskal-Wallis tests according to normality. Individual variables were assessed for homogeneity of variance using Levene’s test. Categorical variables were compared using Pearson’s χ^2^ test or Fisher’s exact test, as appropriate. Relationship between different variables were assessed by correlation analysis: Pearson’s method for normally distributed variables and Spearman’s method for skewed variables.

A receiver operating characteristic (ROC) curve analysis was performed to assess the discriminative power of the different measures of LA function and volumes, LVEF, E/e’ ratio, septal e’, AVA, tricuspid annular plane systolic excursion (TAPSE), or right ventricle/right atrium (RV/RA) gradient. We compared ROC curves using the Delong method.

Survival rates were estimated using Kaplan-Meier method and compared with 2-sided log-rank test. Date of entry into the study was defined as the date of diagnosis (index transthoracic echocardiogram). Univariate and multivariable analysis were used to identify predictors of outcome using Cox proportional hazard models. We used clinically relevant risk-adjusting variables (very severe AS, surgical or percutaneous treatment, age at diagnosis, gender, body mass index (BMI), arterial hypertension, CAD, diabetes, history of malignancy, chronic lung disease, chronic kidney disease, previous symptomatic stroke/transient ischemic attack, Katz index of independence ≤4, dementia, AF appearance during follow-up, LVEF, right ventricular enlargement and TAPSE) in order to adjust for differences in baseline characteristics. Age at diagnosis, LVEF, TAPSE and BMI were processed as continuous variables in Cox hazard model. Risk was expressed as hazard ratio (HR) and their 95% confidence interval (95% CI). In order to check whether Cox model assumptions were respected, we used statistics based on Schoenfeld residuals. To assess nonlinearity, we used Martingale residuals. In every Cox model, we tested for first-order interactions between covariables and each prognosis variable being tested.

Analysis was conducted using IBM® SPSS® Statistics (SPSS for Windows, version 26.0, Armonk, New York) and MedCalc® statistical software (MedCalc software for Windows, version 19.2.0, Ostend, Belgium). All reported *p*-values were 2-sided and *P* values < 0.05 were considered statistically significant.

### Interobserver and intraobserver variability

Interobserver and intraobserver variability of LA volumetric assessment were assessed with the Bland-Altman method [27]. Detailed analysis is depicted in Supplementary Table 1 and Fig. S1.

## Results

### Baseline characteristics

A total of 667 patients were initially enrolled. We excluded 132 patients with AF at index echocardiography, 15 for previous cardiac surgery, 4 for dynamic LVOT obstruction, 16 for unsatisfactory image quality, 49 for having at least mild mitral stenosis, 18 for severe mitral regurgitation, 4 for severe tricuspid regurgitation and 21 for severe aortic regurgitation. After exclusion, a total of 408 patients were included in the analysis. Median follow-up time was 45 months (IQR 54 months). There were no patients lost to follow-up.

The median (IQR) LAEF values were 47 (26) % for the entire cohort, 25 (11) % for patients with LAEF < 37%, 45 (8) % for patients with LAEF 37–53% and 61 (9) % for patients with LAEF ≥54%; median (IQR) LAPEF values were 24 (14) % for the entire cohort, 13.5 (7) % for patients with LAPEF < 19%, 23 (5) % for patients with LAPEF 19–28% and 34 (8) for patients with LAPEF ≥29%; median (IQR) LAAEF values were 29 (27) % for the entire cohort, 10 (9) % for patients with LAAEF < 17%, 27 (9) % for patients with LAAEF 17 to 34% and 46 (13) % for patients with LAAEF ≥35%.

Clinical and demographic characteristics of the 408 patients were significantly different between LAEF groups, as presented in Table 1. Patients with LAEF < 37% were older, had lower BSA, a higher degree of dependence on daily activities, higher prevalence of CAD and chronic kidney disease with lower glomerular filtration rate and developed significantly more often AF during follow-up. The same group registered lower systolic blood pressure at AS diagnosis, lower hemoglobin values and higher international normalized ratios. Regarding drug use by at the moment of AS diagnosis, a higher percentage of patients in the LAEF < 37% group was on diuretics and had insulin-dependent diabetes mellitus. Regarding AVR procedures, there were no significant differences in the type of surgery performed (AVR alone, AVR and coronary artery bypass grafting, AVR and aortic replacement or percutaneous AVR), cardiopulmonary bypass and aortic cross-clamping duration and AVR peri-procedural complications between groups. Detailed information regarding AVR procedures can be seen in Table 1.

**Table 1:** Baseline clinical, analytical and demographic characteristics, global and stratified by left atrial emptying fraction

Variable	All patients (***n*** = 408)	LAEF < 37% (***n*** = 116)	LAEF 37–53% (***n*** = 139)	LAEF ≥ 54% (***n*** = 153)	***P*** Value
Male gender, *n* (%)	221 (53.5)	51 (44.0)	74 (53.2)	94 (61.4)	0.017
Age, years	74.4 ± 10.3	78.7 ± 9.8	75.2 ± 8.4	70.3 ± 1079	< 0.001
Body surface area, m^2^	1.78 ± 0.18	1.73 ± 0.17	1.77 ± 0.19	1.83 ± 0.17	< 0.001
Hypertension, *n* (%)	308 (76.6)	83 (74.1)	100 (73.0)	121 (81.8)	0.167
AF during follow-up, *n* (%)	62 (15.3)	35 (30.7)	17 (12.3)	7 (4.7)	< 0.001
Coronary artery disease, *n* (%)	136 (45.8)	35 (59.3)	45 (41.7)	55 (42.6)	0.061
Dyslipidemia, *n* (%)	268 (66.8)	58 (51.8)	95 (69.9)	112 (75.7)	< 0.001
Diabetes mellitus, *n* (%)	126 (31.3)	38 (33.9)	44 (32.1)	43 (29.1)	0.690
Chronic kidney disease, *n* (%)	168 (41.5)	63 (54.3)	65 (46.8)	39 (26.4)	< 0.001
Stage 5 CKD, *n* (%)	15 (3.8)	5 (4.3)	5 (3.6)	5 (3.5)	0.929
Terminal neoplasia, *n* (%)	6 (1.5)	2 (1.8)	3 (2.2)	1 (0.7)	0.583
Hepatic cirrhosis, *n* (%)	7 (1.8)	0 (0.0)	5 (3.6)	2 (1.4)	0.087
Previous malignancy, *n* (%)	63 (15.7)	19 (17.0)	26 (19.0)	18 (12.2)	0.270
Chronic lung disease, *n* (%)	36 (9.0)	14 (12.5)	13 (9.5)	9 (6.1)	0.199
Previous stroke/TIA, *n* (%)	41 (10.2)	14 (12.5)	9 (6.6)	18 (12.2)	0.202
Katz index ≤4*, *n* (%)	64 (15.9)	32 (28.3)	17 (12.4)	13 (8.8)	< 0.001
Dementia, *n* (%)	22 (5.5)	10 (8.8)	6 (4.4)	5 (3.4)	0.129
GFR, mL/min/1.73 m12	66 ± 28	57 ± 26	65 ± 28	76 ± 27	< 0.001
Systolic blood pressure, mmHg	131 ± 19	129 ± 22	128 ± 16	137 ± 19	0.001
Diastolic blood pressure, mmHg	69 ± 14	68 ± 16	68 ± 14	72 ± 13	0.083
Heart rate, beats per minute	75 ± 15	78 ± 17	75 ± 14	74 ± 14	0.149
Sodium, mmol/L	139 ± 3.0	138 ± 3.9	139 ± 2.7	140 ± 2.3	0.001
Creatinine, mg/dL	1.25 ± 1.07	1.36 ± 1.01	1.30 ± 1.2	1.11 ± 0.96	0.123
Hemoglobin, g/dL	12.7 ± 3.2	11.9 ± 1.7	12.9 ± 4.9	13.1 ± 1.7	0.006
Aspartate aminotransferase, IU/L	26 ± 16	27 ± 11	28 ± 21	24.1 ± 13	0.106
Alanine aminotransferase, IU/L	25 ± 22	27 ± 24	23 ± 17	25 ± 21	0.276
Alkaline phosphatase, IU/L	83 ± 50	93 ± 41	79 ± 28	80 ± 68	0.077
Gamma-glutamyltransferase, IU/L	44 ± 62	55 ± 77	38 ± 47	41 ± 60	0.107
Total bilirubin, mg/dL	0.9 ± 3.3	0.7 ± 0.4	0.7 ± 0.4	1.3 ± 5.3	0.344
International normalized ratio	1.2 ± 0.4	1.3 ± 0.5	1.2 ± 0.5	1.1 ± 0.1	0.001
Antiplatelet, *n* (%)	177 (49.6)	57 (54.8)	57 (46.3)	63 (48.5)	0.424
Statin, *n* (%)	238 (66.7)	64 (61.5)	84 (68.3)	90 (69.2)	0.414
ACEi or ARB, *n* (%)	228 (63.7)	69 (65.7)	70 (56.9)	89 (68.5)	0.142
Calcium-channel blocker, *n* (%)	75 (21.0)	18 (17.3)	23 (18.7)	34 (26.2)	0.189
MRA, *n* (%)	29 (8.1)	11 (10.6)	13 (10.6)	5 (3.8)	0.082
Diuretic, *n* (%)	244 (68.2)	80 (76.2)	87 (70.7)	77 (59.2)	0.016
Oral antidiabetic, *n* (%)	87 (24.3)	22 (21.2)	30 (24.2)	35 (26.9)	0.593
Beta-blocker, *n* (%)	154 (43.1)	51 (49.0)	50 (40.7)	53 (40.8)	0.353
Insulin, *n* (%)	49 (13.7)	22 (21.2)	13 (10.5)	14 (10.8)	0.031
Surgical AVR only, *n* (%)Ŧ	154 (65.8)	18 (54.5)	64 (71.1)	39 (64.9)	0.220
AVR and aorta replacement, *n* (%)Ŧ	15 (6.4)	3 (9.1)	4 (4.4)	8 (7.2)	0.579
AVR and CABG, *n* (%)Ŧ	45 (19.2)	6 (18.2)	15 (16.7)	24 (21.6)	0.666
Percutaneous AVR, *n* (%)Ŧ	18 (7.7)	5 (15.2)	7 (7.8)	6 (5.4)	0.182
AVR bypass time, minutes	71.1 ± 25.7	66.6 ± 16.3	68.9 ± 30.5	74.3 ± 23.3	0.235
AVR aortic clamping time, minutes	42.2 ± 11.4	40.5 ± 9.9	41.9 ± 11.6	42.9 ± 11.8	0.618
AVR procedural complications, *n* (%)Ŧ	10 (4.4%)	0 (0.0)	5 (5.7)	5 (4.6)	0.421

*ACEi* denotes angiotensin-converting-enzyme inhibitor, *AF* atrial fibrillation, *ARB* angiotensin receptor blocker, *AVR* aortic valve replacement, *CABG* coronary artery bypass grafting, *CKD* chronic kidney disease, *GFR* glomerular filtration rate (using Modification of Diet in Renal Disease formula), *IU* international units, *MRA* mineralocorticoid receptor antagonist, *TIA* transient ischemic attack.

* Katz index of independence in activities of daily living, ranging from 6 (patient independent) to 0 (patient very dependent)

Ŧ Percentage relative to patients that were submitted to AVR.

Regarding echocardiographic variables, patients with LAEF < 37% registered lower values in all LA volumetric measures, had lower LVEF, lower AVA and AVAi, greater RV disfunction, higher pulmonary pressures and greater degree of diastolic disfunction with higher LV filling pressures and more commonly shown diastolic L wave. Differences in echocardiographic measures between groups can be seen in Table 2.

**Table 2:** Echocardiographic parameters, global and stratified by left atrial emptying fraction

Variable	All patients (***n*** = 408)	LAEF < 37% (***n*** = 116)	LAEF 37–53% (***n*** = 139)	LAEF ≥ 54% (***n*** = 153)	***P*** Value
LAPEF, %	24.6 ± 10.3	15.7 ± 7.5	25.0 ± 7.9	30.8 ± 9.2	< 0.001
LAPEVi, mL/m^2^	11.2 ± 5.2	9.0 ± 5.2	12.0 ± 4.9	12.2 ± 4.9	< 0.001
LAAEF, %	28.9 ± 16.8	10.2 ± 6.8	26.9 ± 9.3	44.3 ± 11.8	< 0.001
LAAEVi, mL/m^2^	9.4 ± 5.0	5 ± 3.5	9.7 ± 4.1	12.1 ± 4.7	< 0.001
LVEF, %	57.5 ± 11.2	50.8 ± 14.8	59.0 ± 8.6	61.5 ± 6.6	< 0.001
LVEF ≥50%, *n* (%)	344 (83.7)	71 (61.7)	126 (90.6)	145 (95.4)	< 0.001
Aortic valve area, cm^2^	0.75 ± 0.33	0.63 ± 0.20	0.75 ± 0.19	0.84 ± 0.47	< 0.001
Aortic valve indexed to BSA, cm^2^/m^2^	0.44 ± 0.37	0.38 ± 0.11	0.42 ± 0.10	0.51 ± 0.57	0.018
Transaortic mean pressure gradient, mmHg	49.1 ± 14.2	47.9 ± 16.8	49.7 ± 13.7	49.4 ± 12.4	0.562
Peak aortic jet velocity, m/s	4.45 ± 0.59	4.36 ± 0.76	4.47 ± 0.54	4.49 ± 0.48	0.153
Very severe aortic stenosis*, *n* (%)	97 (23.7)	28 (24.6)	33 (23.9)	34 (22.2)	0.894
LVOT VTI / AV VTI ratio	0.23 ± 0.07	0.21 ± 0.06	0.22 ± 0.06	0.25 ± 0.07	< 0.001
Enlarged right ventricle, n (%)	55 (13.3)	30 (25.9)	11 (7.9)	14 (9.2)	< 0.001
TAPSE, mm	21.1 ± 3.9	19.6 ± 4.0	21.5 ± 3.7	22.0 ± 3.8	< 0.001
TAPSE < 16 mm, n (%)	23 (5.6)	16 (13.9)	2 (1.4)	4 (2.6)	< 0.001
RV/RA gradient, mmHg	30.9 ± 11.3	36.3 ± 13.3	26.7 ± 7.1	28.3 ± 9.2	< 0.001
SPAP, mmHg	35.2 ± 13.2	42.0 ± 15.9	30.1 ± 7.6	32.3 ± 10.8	< 0.001
Left ventricular hypertrophy, *n* (%)	222 (55.2)	74 (64.9)	79 (58.1)	67 (45.6)	0.006
Severe left ventricular hypertrophy, *n* (%)	9 (2.2)	6 (5.3)	2 (1.5)	1 (0.7)	< 0.001
Mitral E velocity, m/s	0.83 ± 0.25	0.94 ± 0.25	0.81 ± 0.25	0.79 ± 0.22	< 0.001
Mitral A velocity, m/s	0.96 ± 0.33	0.83 ± 0.43	1.03 ± 3.02	0.99 ± 0.3	< 0.001
Septal e’ velocity, cm/s	4.81 ± 1.70	0.38 ± 0.12	0.47 ± 0.17	0.55 ± 0.16	< 0.001
Septal a’ velocity, cm/s	7.10 ± 2.23	0.51 ± 0.18	0.71 ± 0.18	0.84 ± 0.19	< 0.001
E/A ratio	1.04 ± 0.80	1.59 ± 1.30	0.86 ± 0.41	0.84 ± 0.37	< 0.001
E/e’ ratio	19.22 ± 7.82	26.38 ± 9.33	18.43 ± 5.66	15.17 ± 4.42	< 0.001
Diastolic L wave, *n* (%)	20 (5.9)	14 (16.5)	3 (2.6)	2 (1.5)	< 0.001

*AV* denotes aortic valve, *LAEF* left atrial emptying fraction, *LAAEF* left atrial active emptying fraction, *LAAEVi* indexed left atrial active emptying volume, *LAPEF* left atrial passive emptying fraction, *LAPEVi* indexed left atrial passive emptying volume, *LVEF* left ventricular ejection fraction, *LVOT* left ventricular outflow tract, *RV/RA* right ventricle/right atrium, *SPAP* systolic pulmonary artery pressure, *TAPSE* tricuspid annular plane systolic excursion, *VTI* velocity time integral.

* Defined as transaortic mean pressure gradient ≥60 mmHg or peak aortic jet velocity ≥ 5 m/s

### Clinical outcomes

A total of 185 (44.9%) out of 412 patients died during follow-up. Among the 95 patients with known cause of death, 51 (53.7%) died of cardiac cause (HF, cardiogenic shock, arrhythmias or cardiac arrest not due to non-cardiac causes). The majority of patients in tercile 1 died of cardiac cause, as opposed to patients in tercile 2 and tercile 3 (62.3% versus 46.9 and 22.2%, respectively, *p* = 0.050). A total of 235 patients (57.9%) underwent AVR, the vast majority surgical AVR (92.3%). Cumulative incidence of AVR during follow-up was greater in tercile 2 and 3 (65.2 and 73.8% versus 30.7%, respectively, *P* < 0.001). Among the 235 patients referred to AVR, 30 (8.1%) had depressed LVEF, 6 (7.8% of available information) had > 50 mmHg of systolic pulmonary artery pressure and 59 (25.3%) had very severe AS. A total of 132 (77.2%) of 171 patients that did not undergo AVR died during follow-up. On the other hand, 50 (21.3%) of 235 patients that underwent AVR died during follow-up.

### Outcome impact of LA reservoir function

**Fig. 1:**
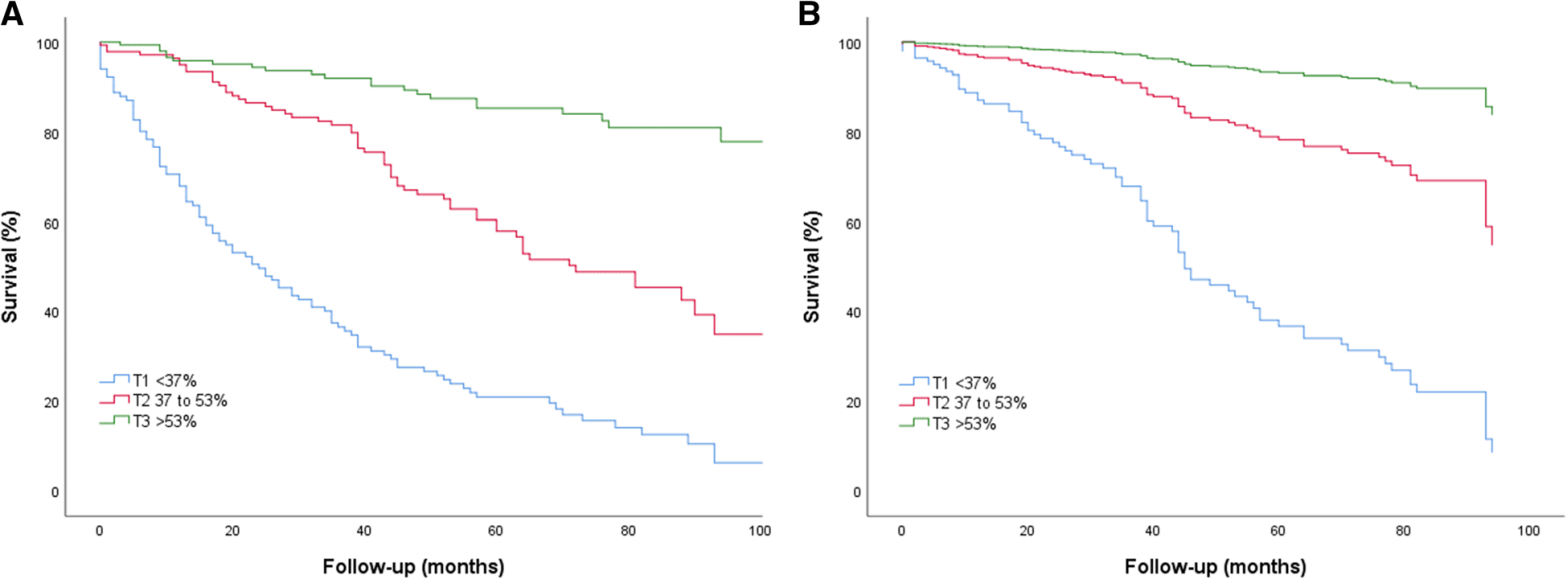
**a**, Kaplan-Meier curves of patients with severe aortic stenosis according to left atrial emptying fraction (LAEF) terciles. **b**, Adjusted survival curves of patients with severe aortic stenosis according to LAEF terciles. Adjustment variables can be seen in Statistics section. T1, T2 and T3 indicate first, second and third tercile

Mean total LA emptying volume was 35.6 (± 13.0) mL, mean indexed total LA emptying volume was 20.1 (± 6.8) mL/m^2^ and mean LAEF was 45.9 (± 16.3) %. Survival during follow-up was progressively higher with increasing LAEF from LAEF < 37% to LAEF ≥54% (14.7, 56.8 and 85.5%, respectively, *P* < 0.001). Patients with LAEF < 37% had a significantly higher all-cause mortality during follow-up compared with patients with LAEF ≥37% (85.3% versus 28.2%, *P* < 0.001; Fig. 1a). After adjustment for clinical and demographic variables, cumulative survival of patients with LAEF < 37% and LAEF 37 to 53% compared to patients with LAEF ≥54% remained significantly lower (adjusted HR 13.91, 95% CI 6.20–31.19, *P* < 0.001 and adjusted HR 3.40, 95% CI 1.57–7.37, *P* = 0.002, respectively; Table 3). Survival was also higher in patients with LAEF 37 to 53% when compared to patients with LAEF < 37% (adjusted HR 0.25, 95% CI 0.14–0.43, *P* < 0.001). When treated as a continuous variable, LAEF remained independently associated with all-cause mortality, with a significant increase in survival with increasing LAEF (adjusted HR 0.92, 95% CI 0.90–0.94, per % increase, *P* < 0.001).

**Table 3:** Relative risk of all-cause mortality associated with left atrial functional assessment

Variable	All-cause mortality	
	HR (95% CI)	***P*** value
**LAEF**		
Unadjusted		
> 53%	Reference	
37–53%	3.57 (2.19–5.83)	< 0.001
< 37%	11.18 (7.01–17.82)	< 0.001
Model 1*		
> 53%	Reference	
37–53%	3.40 (1.57–7.37)	0.002
< 37%	13.91 (6.20–31.19)	< 0.001
Model 2^Ŧ^		
> 53%	Reference	
37–53%	3.59 (1.65–7.80)	0.001
< 37%	11.71 (5.20–26.40)	< 0.001
**LAPEF**		
Unadjusted		
> 28%	Reference	
19–28%	1.64 (1.07–2.51)	0.025
< 19%	3.72 (2.46–5.61)	< 0.001
Model 1*		
> 28%	Reference	
19–28%	1.58 (0.81–3.08)	0.178
< 19%	3.07 (1.60–5.90)	0.001
Model 2^Ŧ^		
> 28%	Reference	
19–28%	1.43 (0.74–2.76)	0.286
< 19%	2.29 (1.16–4.54)	0.018
**LAAEF**		
Unadjusted		
> 34%	Reference	
17–34%	3.27 (1.93–5.54)	< 0.001
< 17%	8.21 (4.98–13.53)	< 0.001
Model 1*		
> 34%	Reference	
17–34%	1.68 (0.77–3.68)	0.196
< 17%	4.51 (1.99–10.19)	< 0.001
Model 2^Ŧ^		
> 34%	Reference	
17–34%	1.97 (0.89–4.37)	0.094
< 17%	4.46 (1.92–10.36)	< 0.001

*CI* denotes confidence interval, *HR* hazard ratio, *LAAEF* left atrial active emptying fraction, *LAEF* left atrial emptying fraction, *LAPEF* left atrial passive emptying fraction.

*Model 1 was adjusted for very severe aortic stenosis, age at diagnosis, gender, body mass index (BMI), arterial hypertension, chronic kidney disease, coronary artery disease, diabetes, history of malignancy, chronic lung disease, previous symptomatic stroke/transient ischemic attack, Katz index of independence ≤4, dementia, atrial fibrillation appearance during follow-up, *LVEF* right ventricular enlargement and TAPSE

ŦModel 2 was adjusted for variables used in model 1 and aortic valve replacement during follow-up.

All associations remained true after adjustment for AVR, as LAEF impact on mortality during follow-up was still significant (LAEF < 37% versus LAEF 37 to 53% and LAEF ≥54%, respectively, adjusted HR 3.59, 95% CI 1.65–7.80, *P* = 0.001 and adjusted HR 11.71, 95% CI 5.20–26.40, *P* < 0.001, respectively; Fig. 1b). The incidence of all-cause mortality when starting follow-up at time of AVR still tended to be higher in patients with LAEF < 37% when compared to patients with LAEF 37 to 53% and patients with LAEF ≥54% (60.0% versus 26.7 and 4.5%, *P* < 0.001).

**Fig. 2:**
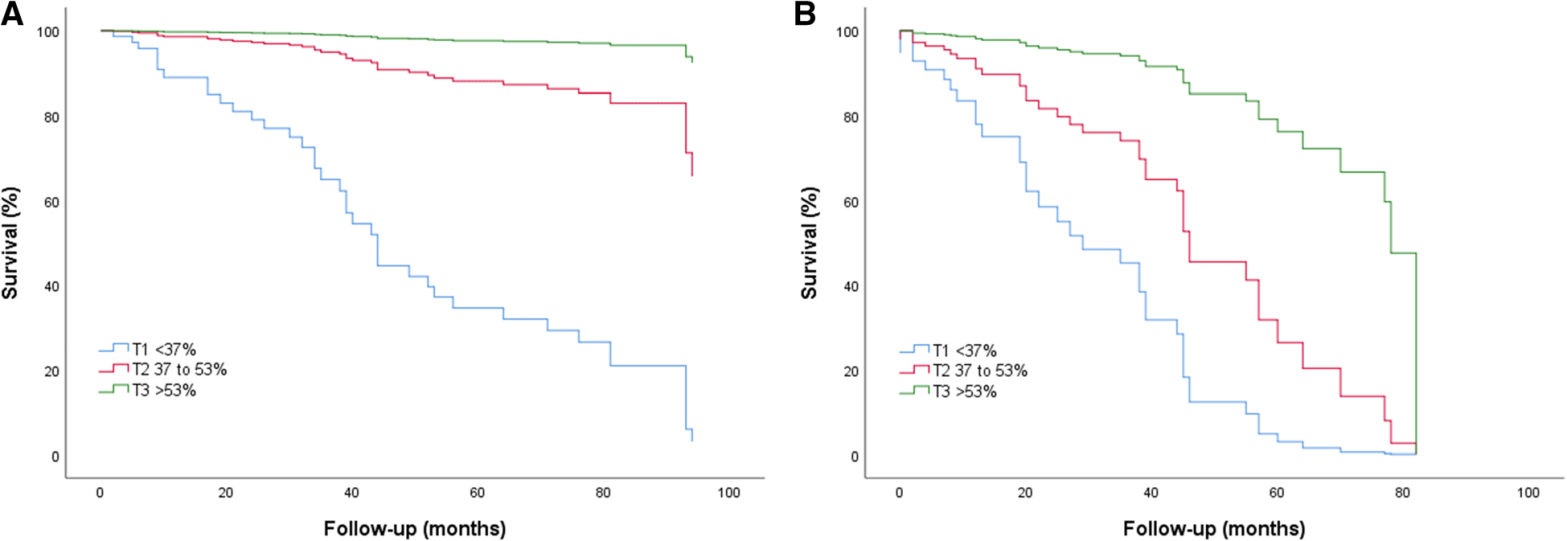
**a**, Adjusted survival curves of patients with severe aortic stenosis according to LAEF terciles in patients who underwent aortic valve replacement. **b**, Adjusted survival curves of patients with severe aortic stenosis according to LAEF terciles in patients who did not underwent aortic valve replacement. Adjustment variables can be seen in Statistics section. T1, T2 and T3 indicate first, second and third tercile

As we found AVR to interact with different terciles of LAEF, we performed a separate analysis for patients that underwent AVR and patients who did not underwent AVR. We found that LAEF was still a potent predictor of all-cause mortality in the case of patients who underwent AVR (LAEF < 37% versus LAEF ≥54%, adjusted HR 44.61, 95% CI 13.36–147.79, *P <* 0.001) and in the case of patients who did not underwent AVR (adjusted HR 12.99, 95% CI 2.97–56.96, *P* = 0.001), as shown in Fig. 2.

### Subgroup analysis

We found no interaction between age at diagnosis, BSA, BMI, arterial hypertension, presence of CAD, diabetes mellitus, previous symptomatic stroke/transient ischemic attack, Katz index of independence ≤4, AF appearance during follow-up, LVEF, right ventricular enlargement, TAPSE, very severe AS, E/e’, septal e’ velocity and the outcome impact of different LAEF terciles (all variables *P* for interaction > 0.05). As we found gender to interact with different terciles of LAEF, we performed a separate analysis for each gender, and we observed that predictive power of different terciles was greater in women (LAEF < 37% versus LAEF ≥54% in women, HR 15.47, 95% CI 8.15–30.25, *P* < 0.001; LAEF < 37% versus LAEF ≥54% in males, HR 8.59, 95% CI 3.00–23.84, *P* < 0.001). Mortality during follow-up was progressively higher as AVA was smaller, both in LAEF < 37% (AVA > 0.8 cm^2^, 81.5%, AVA 0.6 to 0.8 cm^2^, 82.5% and AVA < 0.6 cm^2^, 94.7%, *P* = 0.185) and LAEF ≥37% (AVA > 0.8 cm^2^, 23.0%, AVA 0.6 to 0.8 cm^2^, 29.5% and AVA < 0.6 cm^2^, 46.2%, *P* = 0.044). In patients with preserved LVEF, mortality during follow-up was consistently higher in patients with LAEF < 37% compared with patients with LAEF ≥37% (90.1% versus 26.7%, *P* < 0.001) as well as in patients with depressed LVEF (77.3% versus 45.0%, *P* = 0.011). The impact of LAEF < 37% in prognosis was still observed in patients with LVH (90.5% versus 33.8%, *P* < 0.001) and without LVH (75.0% versus 20.4%, *P* < 0.001).

### Outcome impact of LA conduit and pump functions

A detailed analysis of outcome impact of LA conduit and pump functions can be seen in Supplementary material.

### Comparison between LA volumetric parameters and other echocardiographic features as predictors of mortality

**Fig. 3:**
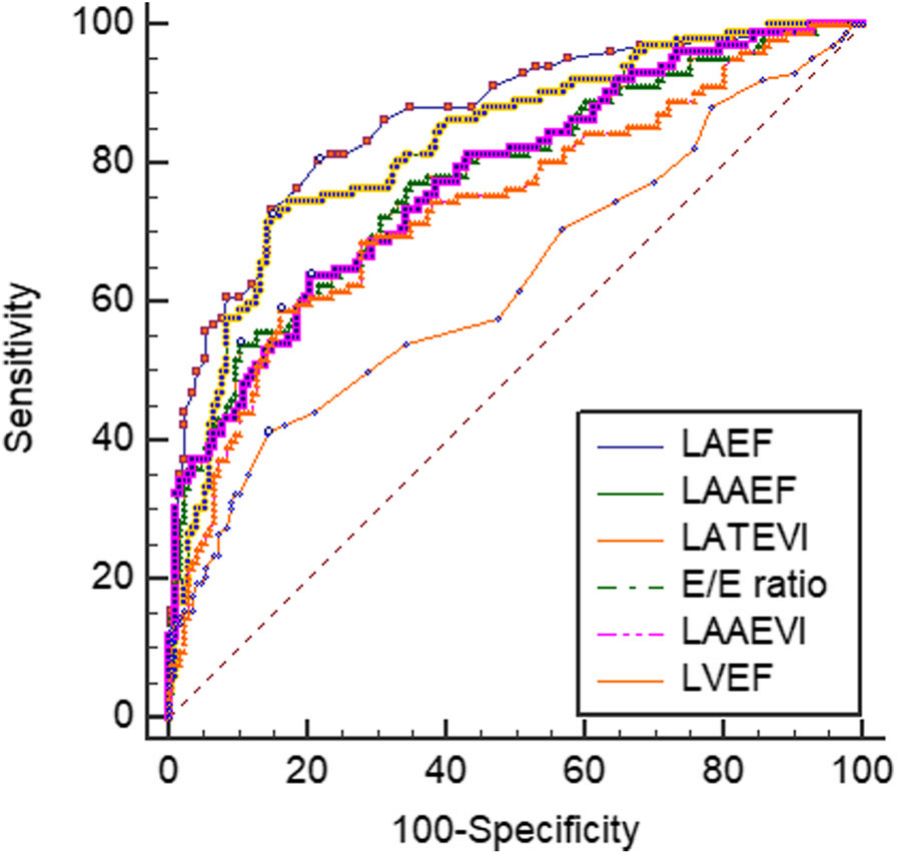
ROC analysis curves of LAEF, LAAEF, LATEVi, E/e’ ratio, LAAEVI and LVEF for all-cause mortality. LAAEF, left atrial active emptying fraction; LAAEVi, indexed left atrial active emptying volume; LAEF, left atrial emptying fraction; LATEVi, indexed left atrial total emptying volume; LVEF, left ventricular ejection fraction

We entered different LA volumetric parameters into ROC analysis in order to estimate the probability of death at follow-up. Different variables and respective AUCs are depicted in Table 4 and Fig. 3. Compared to classical echocardiographic AS severity parameters (V_max_, AVA and MPG), LAEF at AS diagnosis emerged as the best discriminator of all-cause mortality. Between LA volumetric parameters, LAEF also persisted as the most accurate predictor of mortality during follow-up (AUC 0.845), which was greater than LAPEF (0.845 versus 0.709, *P* < 0.001), LAAEF (0.845 versus 0.812, P < 0.001), indexed LA total emptying volume (0.845 versus 0.772, *P* < 0.001), indexed LA passive emptying volume (0.845 versus 0.626, *P* < 0.001) and indexed LA active emptying volume (0.845 versus 0.723, *P* < 0.001). Optimal cut-off values to identify higher mortality were 37% for LAEF, 19% for LAPEF and 23% for LAAEF. The best cut-off value of LAEF associated with increased mortality had a sensitivity of 56% and a specificity of 92%.

**Table 4:** Discriminative power of echocardiographic parameters for all-cause mortality

Variables	AUC	95% CI	***P-value***	***Sensitivity (%)***	***Specificity (%)***	***Criterion***
LAEF (%)	0.845	0.807–0.879	< 0.001	56	92	37
LAAEF (%)	0.812	0.768–0.850	< 0.001	71	82	23
LATEVi (mL)	0.783	0.738–0.824	< 0.001	60	84	17
E/e’ ratio	0.772	0.721–0.817	< 0.001	63	79	20
Septal e’ velocity (cm/s)	0.758	0.706–0.804	< 0.001	71	67	4
LAAEVi (mL)	0.723	0.672–0.770	< 0.001	57	80	7
LAPEF (%)	0.709	0.660–0.755	< 0.001	42	89	17
AVA (cm^2^)	0.661	0.612–0.709	< 0.001	67	57	0.7
RV/RA gradient (mmHg)	0.627	0.556–0.693	0.001	42	77	32
LAPEVi (mL)	0.626	0.572–0.678	< 0.001	49	71	9
LAVi (mL)	0.625	0.574–0.674	< 0.001	67	56	44
LVEF (%)	0.613	0.564–0.660	< 0.001	42	79	56
TAPSE (mm)	0.573	0.523–0.621	0.010	64	47	21
Aortic MPG (mmHg)	0.555	0.505–0.604	0.058	16	96	34
V_max_ (m/s)	0.550	0.500–0.599	0.085	38	72	4.2

*AUC* denotes area under the curve, *AVA* aortic valve area, *LAAEF* left atrial active emptying fraction, *LAAEVi* indexed left atrial active emptying volume, *LAEF* left atrial emptying fraction, *LAPEF* left atrial passive emptying fraction, *LAPEVi* indexed left atrial passive emptying volume, *LATEVi* indexed left atrial total emptying volume, *LAVi* indexed left atrial maximum volume, *LVEF* left ventricular ejection fraction, *MPG* mean pressure gradient, *TAPSE* tricuspid annular plane systolic excursion, *RV/RA* right ventricle/right atrium.

## Discussion

In the present study, we focused on the impact of LA function on outcome in patients with severe AS. To the best of our knowledge, this is the first study to extensively evaluate the prognostic value of LA functional assessment using classic volumetric parameters in current era severe AS patients. We found that LA function, evaluated by echocardiographic volumetric parameters, is a strong predictor of all-cause mortality, not only in patients who underwent AVR but also in patients who remained on conservative treatment. Also, we observed that in the lower terciles of LAEF mortality was predominantly of cardiac cause when compared to other terciles where less than half of patients died of non-cardiac causes, a finding that reflects the potency of reduced LAEF as a predictor of prognosis attributable to AS itself, mainly in an older population susceptible to die from non-cardiac causes. When compared to other classical prognostic factors in AS such as AVA, V_max_, MPG and LVEF, LA function was a better predictor of mortality during follow-up. Also, this effect of LA function on prognosis remains true even after adjustment for other factors that are known to affect prognosis in AS, such as age, LVEF, AVA and other comorbidities. Diminished LAEF (LAEF ≤53%) was associated with increased risk of all-cause death during follow-up, and was the best echocardiographic predictor in our study. Other LA functional parameters were still powerful predictors of adverse outcome, mainly LAAEF. Also, the impact of LA functional assessment remained significant across different AVA and LA volumes, in patients with preserved or depressed left ventricular systolic function and different degrees of left ventricular hypertrophy. Additionally, our results show that LA functional parameters remain good predictors of mortality after AVR, despite the important impact of the procedure on mortality of these patients. We also found that LA functional assessment is reproducible, easy and fast to obtain, as different phasic volumes were measured by the biplane method of disks, routinely used in the majority of echocardiography laboratories and the recommended method of measuring left atrial volume [28]. Thus, based on the aforementioned findings, we suggest that LA functional evaluation should be performed in all patients evaluated for severe AS and the results should be taken into consideration for the management of these patients.

The background of chronically increased left ventricular afterload in AS is associated with structural and functional changes in the LA. LA enlargement, the most common macroscopic LA structural change, has been considered the most direct noninvasive proof of increased LV filling pressure and diastolic dysfunction [6, 7]. Also, it has been recently associated with higher mortality, even after AVR [18, 29]. Besides LA dilatation, the ongoing pressure overload leads to disturbance in the LA three functional phases: reservoir, conduit and contractile phase [8], particularly in the contractile phase. In our study, we found a reduction in all phasic LA performances, when compared to control groups of patients without cardiac disease [8, 14, 30] and the results from NORRE study, in which 371 healthy subjects were enrolled in order to obtain normal ranges for echocardiographic measures of LA function [31]. As reported in previous studies, the intrinsic left atrial myopathic disease can precede visible LA structural changes, being an early marker of increased LV filling pressures [8, 32]. This finding can explain the patients with normal LA volume and depressed LA function found in our cohort, which showed higher echocardiographic measures of diastolic dysfunction such as elevated septal e’ velocity and higher E/e’ ratios. As AS is a disease in which elevated intracavitary pressures play a very important role in its progression, the finding of LA functional change may represent an important milestone in which AVR may play an important role in its modification. As such, LA volume and function best capture the cardiac remodelling associated with AS, contrary to other echocardiographic variables.

While much is known about LA structural damage as a predictor of death in different diseases such as dilated cardiomyopathy [15], myocardial infarction [16], mitral regurgitation [17] and more recently in AS [18], there is limited information regarding LA function as a predictor of prognosis in patients with AS. There have been some reports showing that LA function assessed by speckle-tracking echocardiography can predict worse outcomes in AS patients. In a study conducted in our center, Marques-Alves et al. found that, in a population of patients with moderate and severe AS, LA global strain was the best discriminator of AS severity and a significant predictor of a composite of HF, death and AVR [19]. The same study also found that atrial mechanics were better predictors of prognosis that LV global longitudinal strain, which was not a significant predictor of outcome. In another study by Todaro et al., which recruited 89 asymptomatic patients with severe AS and normal LVEF and 40 age- and gender-matched controls, in which LA and LV mechanics were measured by speckle-tracking echocardiography, LV global longitudinal strain, LA reservoir and LA stiffness were found to be strong predictors of adverse events during follow-up [20]. However, on multivariate analysis only LV global longitudinal strain remained a significant predictor of events recurrence. Galli et al. also found that in a population of 128 patients with severe AS, global peak LA strain measured by speckle-tracking echocardiography was a significant independent predictor major adverse cardiac events [21]. No study has evaluated the impact of volumetric assessment of LA function on outcome of severe AS patients, as in all LA function was assessed by speckle-tracking echocardiography. This technique has some advantages compared to volumetric methods, as it makes no geometric assumptions, does not need to make multiple plane acquisition and is, theoretically, less time consuming. Although the risk of LA foreshortening and the assumption of a geometric model of a non-symmetric chamber are real, we found LA volumetric assessment to be an easy, reproducible and fast method of LA evaluation. Besides, if the intention is to measure LA emptying fractions, the problem of foreshortening and eventual underestimation of LA volumes is less important, as it would not impact on the emptying fraction values. STE also has some limitations, as it is prone to suboptimal tracking of the endocardial border, is sensible to acoustic shadowing and reverberations, is not absolutely angle-independent and relies on good image quality. Moreover, each provider has his own software package and it is not available in every echocardiography laboratory.

Other published studies that addressed the impact of LA function on prognosis used as outcome a composite of HF, death and AVR [19], occurrence of symptoms and death [20] and major adverse cardiac events [21], outcomes that can be broad. In our study, the measured outcome was all-cause mortality, still, the best predictor of outcome found (LAEF representing LA reservoir function) showed very good predictive value. Also, our study had a long follow-up period compared to other studies, which is important not only to reduce immortality time bias but also to better understand the clinical course of AS patients.

### Limitations

Our study had a retrospective design and, as such, has the inherent limitations of such studies. We did not record the specific indications for AVR, however, all decisions for AVR were taken in a heart team with extensive experience in valvular heart disease and who assures good practice according to guidelines. We included only patients with severe AS, so we can not extrapolate our findings to moderate or mild AS or even to other valvular diseases. Also, we excluded all patients with AF at the baseline exam and with previous history of the disease. This criterion excluded many patients from analysis. However, we registered the development of AF during follow-up, which gave us the possibility to evaluate its impact on patient prognosis. Finally, we did not record the reason for conservative management, so we do not know the extent of patient AVR refusal or what led our heart team to make that decision.

## Conclusion

In patients with a first diagnosis of severe AS in hospital setting, LA function assessed by volumetric parameters is an independent predictor of all-cause mortality. Compared to classical severity parameters, different LA functional parameters were found to be more potent predictors of death. These data can be useful in clinical practice for risk stratification and therefore for decision of timing for AVR.

## Supplementary Information

**Additional file 1:.**

## Data Availability

The datasets used and/or analysed during the current study are available from the corresponding author on reasonable request.
