# Supplementary material for: Left atrial functional assessment and mortality in patients with severe aortic stenosis with sinus rhythm
Source: Cardiovasc Ultrasound. 2021 Jan 2;19:1. doi: 10.1186/s12947-020-00231-0 (PMC7778823; doi:10.1186/s12947-020-00231-0)
Supplement: Supplementary file 1 — Additional file 1:. [file 12947_2020_231_MOESM1_ESM.docx]

***Interobserver and intraobserver variability***

Analyses were performed by the same echocardiographer in 20 randomly selected patients, being repeated 2 weeks later in order to determine intraobserver variability. Interobserver variability was assessed in the first group of 20 randomly selected patients by a second echocardiographer (VG), who repeated the analyses, comparing obtained values with the measurements made by the first echocardiographer. Both echographers were blinded to the previous measurements. Detailed analysis is shown in Table S1 and Figure S1.

| **Table S1. Variability of echocardiographic variables (n=20)** | | | |
| --- | --- | --- | --- |
|  | **Interobserver variability** | **Intraobserver variability** | |
|  | **Bias (limit of agreement)** | | **Bias (limit of agreement)** |
| LAEF | -2.1 % (-16.1; 11.9)  -3.0 % (-17.2; 10.4)  -2.7 % (-16.6; 10.2) | | 1.4 % (-7.4; 10.1)  1.9 % (-9.1; 10.5)  1.7 % (-8.6; 10.2) |
| LAPEF |  |  |  |
| LAAEF |  |  |  |
| LAAEF denotes left atrial active emptying fraction; LAEF, left trial emptying fraction; LAPEF, left atrial passive emptying fraction. | | | |


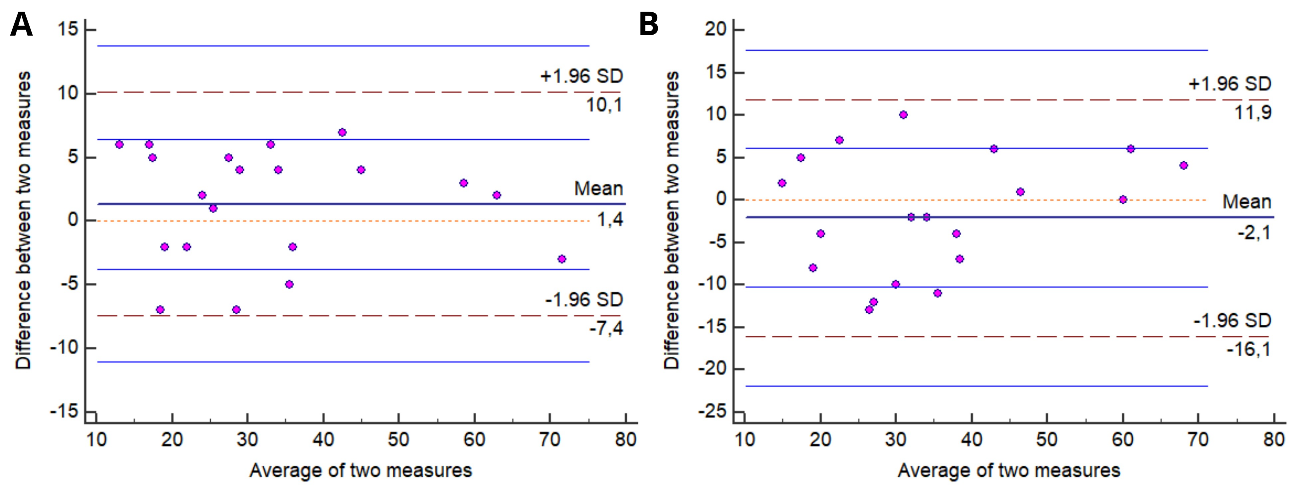


**Figure S1.** A, Bland-Altman plot for intraoperator reproducibility of left atrial emptying fraction. B, Bland-Altman plot for interoperator reproducibility of left atrial emptying fraction.

***Outcome impact of LA conduit function***

Mean LA passive emptying volume was 19.9 (± 9.5) mL, mean indexed LA passive emptying volume was 11.2 (± 5.2) mL/m^2^ and mean LAPEF was 24.6 (± 10.3) %. Survival during follow-up was progressively higher with increasing LAPEF from patients with LAPEF <19% to patients with LAPEF ≥28% (35,8%, 58.1% and 74.0%, respectively, *P*<0.001, Figure S2A). After adjustment for clinical and demographic variables, cumulative survival of LAPEF <19% relative to LAPEF ≥28% remained significantly different (adjusted HR 3.07, 95% CI 1.60-5.90, *P*=0.001; Table 3) but not when patients with LAPEF 19 to 27% were compared to patients with LAPEF ≥28% (adjusted HR 1.58, 95% CI 0.81-3.08, *P*=0.185). Still, survival during follow-up between patients with LAPEF 19 to 27% and patients with LAPEF <19% remained significantly different (adjusted HR 0.50, 95% CI 0.27-0.93, *P*=0.030). When treated as a continuous variable, LAPEF remained independently associated with all-cause mortality, with a significant increase in survival with increasing LAEF (adjusted HR 0.94, 95% CI 0.92-0.97, per % increase, *P*<0.001). All associations remained true even after adjustment for AVR, as LAPEF still impacted mortality during follow up (LAPEF <19% versus LAPEF ≥28%, adjusted HR 2.29, 95% CI 1.16-4.54, *P*=0.018, Figure S2B). However, there was no difference between patients with LAPEF <19 and patients with LAPEF 19 to 27% (adjusted HR 1.43, 95% CI 0.74-2.76, *P*=0.286). The incidence of all-cause mortality when starting follow-up at time of AVR still tended to be higher in LAPEF <19% when compared to LAPEF 19 to 27% and LAPEF ≥28% (30.2% versus 17.9% and 15,6%, respectively, *P*=0.002).


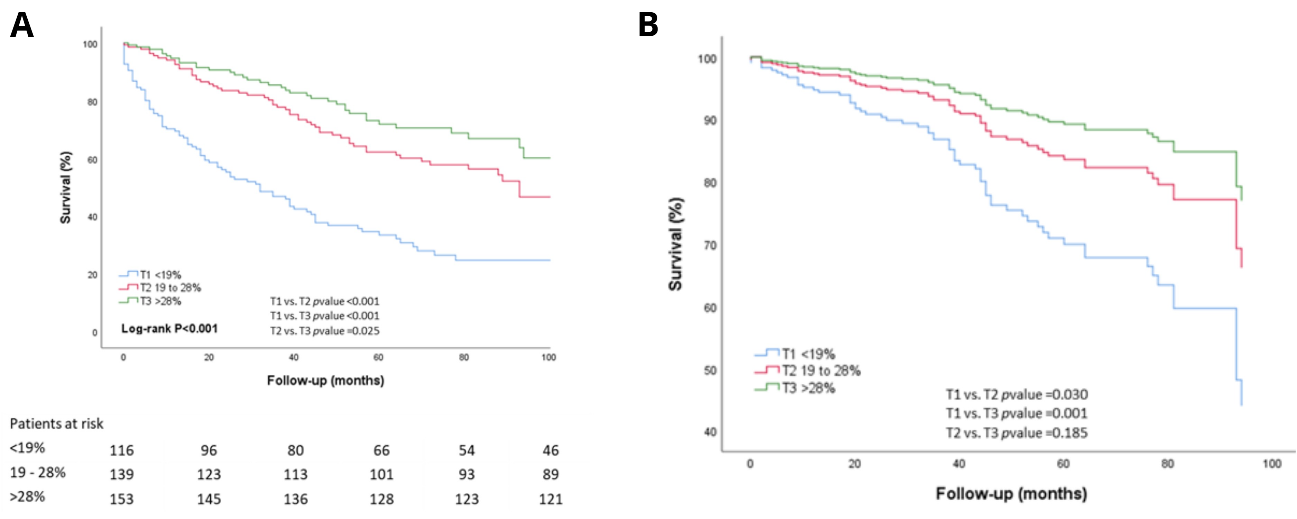


**Figure S2.** A, Kaplan-Meier curves of patients with severe aortic stenosis according to left atrial passive emptying fraction (LAPEF) terciles. B, Adjusted survival curves of patients with severe aortic stenosis according to LAPEF terciles. Adjustment variables can be seen in Statistics section. T1, T2 and T3 indicate first, second and third tercile.

*Subgroup analysis*

We found no interaction between age at diagnosis, gender, BSA, BMI, presence of coronary artery disease, diabetes mellitus, previous symptomatic stroke/transient ischemic attack, Katz index of independence ≤ 4, LVEF, TAPSE, very severe AS, septal e’ velocity and the outcome impact of LAPEF <37% (all variables *P* for interaction >0.05). We found a significant interaction between different terciles of LAPEF and atrial fibrillation development during follow-up (*P*=0.012). We observed that predictive power of different terciles was greater in patients who did not develop AF during follow-up (LAPEF <19% versus LAPEF ≥28%, HR 4.43, 95% CI 2.95-6.37, *P*<0.001) being non-significant in patients who developed AF during follow-up (LAPEF <19% versus LAPEF ≥28%, HR 2.100, 95% CI 0.64-6.35, *P*=0.230). Mortality during follow-up was progressively higher as AVA was smaller, both in LAPEF <19% (AVA>0.8 cm^2^, 45.9%, AVA 0.6 to 0.8 cm^2^, 68.6% and AVA<0.6 cm^2^, 88.5%, *P*<0.001) and LAPEF ≥19% (AVA>0.8 cm^2^, 25.2%, AVA 0.6 to 0.8 cm^2^, 33.3% and AVA<0.6 cm^2^, 61.8%, *P*<0.001). In patients with preserved LVEF, mortality during follow-up was higher in patients with LAPEF <19% compared with patients with LAEF ≥19% (61.3% versus 38.7%, *P*<0.001) but was not significantly different in patients with depressed LVEF (69.7% versus 63.0%, *P*=0.596). The impact of LAPEF <19% in prognosis was still observed in patients with LVH (71.0% versus 41.4%, *P*<0.001) and without LVH (50.0% versus 26.6%, *P*=0.014).

***Outcome of LA pump function***

Mean LA active emptying volume was 16.4 (± 9.4) mL, mean indexed LA emptying volume was 9.4 (± 5.0) mL/m^2^ and mean LAAEF was 28.9 (± 16.8) %. Survival during follow-up was progressively higher with increasing LAAEF from patients with LAAEF <17% to patients with LAAEF ≥35% (22.0%, 59.2% and 85.6%, respectively, *P*<0.001, Figure S3A). After adjustment for clinical and demographic variables, survival at follow-up of patients with LAAEF <17% and patients with LAAEF 17 to 34% was lower when compared to patients with LAAEF ≥35% (adjusted HR 4.51, 95% CI 1.99-10.19, *P*<0.001 and adjusted HR 1.68, 95% CI 0.76-3.68, *P*=0.196, respectively; Table 3). Survival of patients with LAAEF 17 to 34% was also higher when compared to patients with LAAEF <17% (adjusted HR 0.37, 95% CI 0.20-0.69, *P*=0.002). When treated as a continuous variable, LAAEF remained independently associated with all-cause mortality, with a significant increase in survival with increasing LAAEF (adjusted HR 0.95, 95% CI 0.93-0.97, per % increase, *P*< 0.001).

After adjustment for AVR, LAAEF impact on mortality during follow-up was still significant in patients with LAAEF <17% versus patients with LAAEF ≥35% (adjusted HR 4.46, 95% CI 1.92-10.36, *P*<0.001, Figure S3B) but not in patients with LAAEF <17% versus patients with LAAEF 17 to 34% (adjusted HR 1.97, 95% CI 0.89-4.37, *P*=0.094). The incidence of all-cause mortality when starting follow-up at time of AVR still tended to be higher in LAAEF <17% when compared to LAAEF 17 to 34% and LAAEF ≥35% (52.8% versus 17.3% and 8.1%, *P*<0.001).


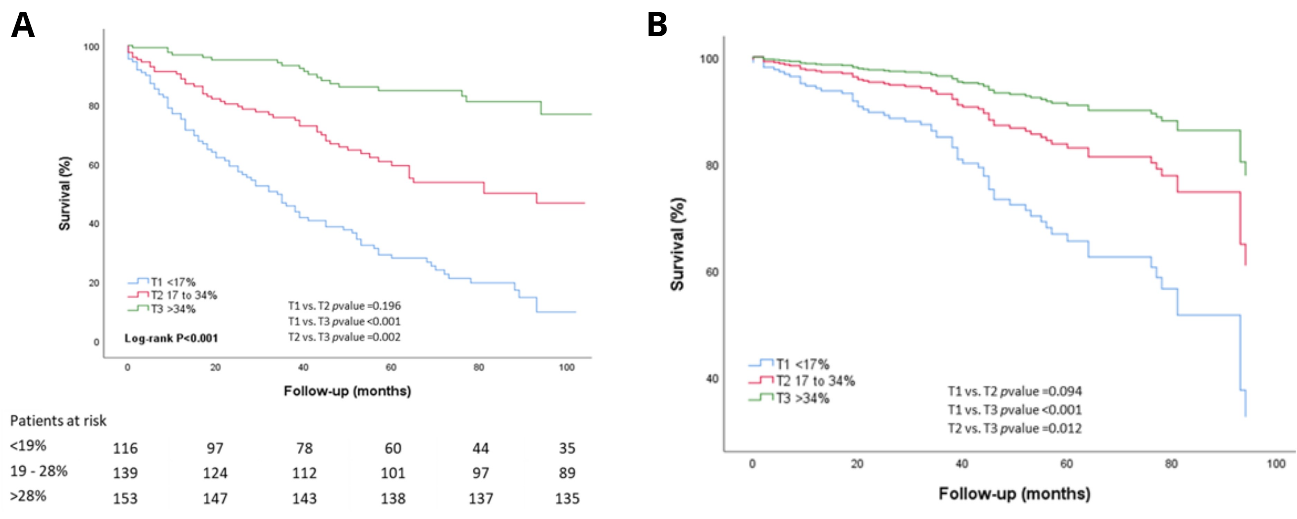


**Figure S3.** A, Kaplan-Meier curves of patients with severe aortic stenosis according to left atrial active emptying fraction (LAAEF) terciles. B, Adjusted survival curves of patients with severe aortic stenosis according to LAAEF terciles. Adjustment variables can be seen in Statistics section. T1, T2 and T3 indicate first, second and third tercile.

*Subgroup analysis*

We found no interaction between age at diagnosis, BSA, BMI, arterial hypertension, presence of coronary artery disease, diabetes mellitus, previous symptomatic stroke/transient ischemic attack, Katz index of independence ≤ 4, atrial fibrillation appearance during follow-up, LVEF, right ventricular enlargement, TAPSE, very severe AS, E/e’, septal e’ velocity and the outcome impact of different LAEF terciles (all variables *P* for interaction >0.05). Mortality during follow-up was progressively higher as AVA was smaller, both in LAAEF <17% (AVA>0.8 cm^2^, 75.0%, AVA 0.6 to 0.8 cm^2^, 71.9% and AVA<0.6 cm^2^, 88.6%, *P*=0.202) and LAEF ≥17% (AVA>0.8 cm^2^, 18.3%, AVA 0.6 to 0.8 cm^2^, 31.8% and AVA<0.6 cm^2^, 52.0%, *P*=0.001). In patients with preserved LVEF, mortality during follow-up was consistently higher in patients with LAAEF <17% compared with patients with LAAEF ≥17% (78.3% versus 25.7%, *P*<0.001) as well as in patients with depressed LVEF (77.5% versus 42.1%, *P*<0.001). The impact in all-cause mortality of LAAEF <17% compared to LAAEF ≥17% was still observed in patients with LVH (83.3% versus 34.9%, *P*<0.001) and without LVH (70.0% versus 18.7%, *P*<0.001).
